# Phase II study of bi-weekly administration of paclitaxel and cisplatin in patients with advanced oesophageal cancer

**DOI:** 10.1038/sj.bjc.6600166

**Published:** 2002-03-04

**Authors:** M B Polee, F A L M Eskens, M E L van der Burg, T A W Splinter, P D Siersema, H W Tilanus, J Verweij, G Stoter, A van der Gaast

**Affiliations:** Department of Medical Oncology, University Hospital Rotterdam-Dijkzigt, Rotterdam, The Netherlands; Department of Gastroenterology, University Hospital Rotterdam-Dijkzigt, Rotterdam, The Netherlands; Department of Surgery, University Hospital Rotterdam-Dijkzigt, Rotterdam, The Netherlands

**Keywords:** advanced oesophageal cancer, chemotherapy, cisplatin, paclitaxel

## Abstract

In a phase I study we demonstrated the feasibility of a bi-weekly combination of paclitaxel 180 mg m^−2^ with cisplatin 60 mg m^−2^. In this study we further assessed toxicity and efficacy of this schedule in the treatment of advanced cancer of the oesophagus or the gastro-oesophageal junction. Patients received paclitaxel 180 mg m^−2^ administered over 3 h followed by a 3-h infusion of cisplatin 60 mg m^−2^. Patients were retreated every 2 weeks unless granulocytes were <0.75×10^9^ or platelets <75×10^9^. Patients were evaluated after three and six cycles and responding patients received a maximum of eight cycles. Fifty-one patients were enrolled into the study. The median age was 56 years (range 32–78). WHO performance status were: 0 (19 patients); 1 (29 patients); 2 (three patients). All patients received at least three cycles of chemotherapy and all were evaluable for toxicity and response. Haematological toxicity consisted of uncomplicated neutropenia grade 3 in 39% and grade 4 in 31% of patients. Five patients (10%) were hospitalised, three patients because of treatment related complications and two patients because of infections without neutropenia. Sensory neurotoxicity was the predominant non-haematological toxicity; grade 1 and 2 neurotoxicity was observed in 43 and 20% of patients, respectively. Response evaluation in 51 patients with measurable disease: complete response 4%, partial response 39%, stable disease 43% and progressive disease in 14% of the patients. The median duration of response was 8 months. The median survival for all patients was 9 (range 2–29+) months and the one-year survival rate was 43%. Four patients who received additional local treatment (two patients surgery and two patients radiotherapy) are still disease free after a follow-up of 20–29 months. This bi-weekly treatment of paclitaxel and cisplatin is well tolerated by patients with advanced oesophageal cancer. The toxicity profile of this regimen compares favourable to that of previously used cisplatin- and paclitaxel-based regimens. Trials are underway evaluating this bi-weekly regimen in a neo-adjuvant setting.

*British Journal of Cancer* (2002) **86**, 669–673. DOI: 10.1038/sj/bjc/6600166 www.bjcancer.com

© 2002 Cancer Research UK

## 

The incidence of oesophageal cancer is rising in the United States and most Northern European countries, especially due to a rapid increase in the incidence of adenocarcinomas of the distal oesophagus or the gastro-oesophageal junction (Blot and McLaughlin, 1999). Although adenocarcinomas are known to be related to symptoms of gastric reflux (Lagergren *et al*, 1999) and to specialised columnar (Barrett's) epithelia (Jankowski *et al*, 1999) it is questionable whether this totally accounts for the rising incidence.

Many patients who present with symptoms of oesophageal obstruction already have locally advanced or metastatic disease. After surgery the 5-year survival is 20% and the majority of patients relapse both locoregionally as well as at distant sites (Millikan *et al*, 1995). Multimodality treatment plays an increasingly important role in the treatment of oesophageal cancer. Chemotherapy with concurrent radiotherapy has been shown to be superior to radiotherapy alone in patients with locoregional disease (Herskovic *et al*, 1992). However, pre-operative treatment with chemotherapy remains still investigational because a number of randomised studies have provided conflicting results (Kok *et al*, 1997; Kelsen *et al*, 1998; Clark, 2000). Chemotherapy can also be given for palliation of symptoms and improvement of quality of life in patients with metastatic disease (Spiridonidis *et al*, 1996; Ilson *et al*, 1999).

Combination chemotherapy with cisplatin and 5-fluorouracil and/or etoposide or with bleomycin and vindesine has predominantly been used in patients with squamous cell carcinoma (Kelsen *et al*, 1990; Kok *et al*, 1996; Bleiberg *et al*, 1997), yielding response rates of 45–75% in patients with locoregional disease and 25–35% in patients (Enzinger *et al*, 1999).

Single agent paclitaxel has been tested in squamous cell and adenocarcinomas of the oesophagus. Ajani *et al* (1994) reported a response rate of 31% after treatment with paclitaxel 250 mg m^−2^ administered every 3 weeks in combination with granulocyte colony-stimulating factor support. In combination with cisplatin, also in a 3-week schedule, Kelsen *et al* (1997) reported a response rate of 49% for patients with either locoregional or metastatic oesophageal cancer.

We previously performed a dose finding study with a fixed cisplatin dose (60 mg m^−2^) and increasing doses of paclitaxel given every 2 weeks in patients with advanced oesophageal cancer (van der Gaast *et al*, 1999). The paclitaxel dose could be increased to 200 mg m^−2^ without encountering dose limiting haematological toxicity. However sensory neurotoxicity was dose limiting at paclitaxel dose levels ⩾190 mg m^−2^. The recommended dose for further studies was paclitaxel 180 mg m^−2^ in combination with cisplatin 60 mg m^−2^. In view of the response rate of 52% observed in this dose finding study we performed a phase II study to further confirm the safety and activity of this bi-weekly regimen.

## MATERIALS AND METHODS

### Patients

Patients with histologically proven metastatic or unresectable adenocarcinoma, undifferentiated- or squamous cell carcinoma of the oesophagus or gastro-oesophageal junction area were eligible for the study. Further eligibility requirements were: a life expectancy of more than 12 weeks; age ⩽18 years; WHO performance status 0–2; written informed consent; adequate haematological, renal and hepatic functions defined as: granulocytes ⩾1.5× 10^9^ l^−1^, platelets ⩾100×10^9^ l^−1^, total bilirubin ⩽1.5×upper normal limit and creatinine ⩽120 μmol l^−1^. Patients were required to have measurable or evaluable disease. Prior radiotherapy was allowed if not involving more than 30% of the bone marrow or was given within the 4 weeks prior to study entry. The study was approved by the institutional ethics committee.

### Drug administration

Paclitaxel 180 mg m^−2^ and cisplatin 60 mg m^−2^ were administered intravenously every 2 weeks. After prehydration with at least 1 l of normal saline, paclitaxel, diluted in 500 ml of normal saline, was infused over 3 h and subsequently cisplatin was administered over 3 h followed by post-hydration over 24 h. All patients received premedication with dexamethasone 20 mg given orally 12 and 6 h prior to the paclitaxel infusion. Thirty minutes before the paclitaxel infusion, the patients received 10 mg dexamethasone, 2 mg clemastine and 50 mg ranitidine, all given i.v. Ondansetron at a dose of 8 mg i.v. was given as anti-emetic prophylaxis. Patients were retreated after 14 days when the granulocytes were ⩾0.75×10^9^ l^−1^ and the platelets were ⩾75×10^9^ l^−1^. When these criteria were not met, treatment was postponed for 1 week. A dose reduction was only made for patients with neutropenic fever; in that case paclitaxel was reduced to 75% in subsequent courses.

### Treatment assessment

Pre-treatment evaluations consisted of a complete medical history, physical examination, complete blood cell count and serum biochemistry, computerised tomography (CT) scan of the chest and upper abdomen and ultrasonography of the supraclavicular nodes when appropriate. Patients with the primary tumour *in situ* were also evaluated by endoscopy. During treatment blood cell counts were assessed every week and physical examination, toxicity assessment and serum chemistry studies every 2 weeks. Toxicity was graded and reported using NCI–CTC criteria (version 2). For response evaluation the CT-scan, and also a ultrasonography and endoscopy when appropriate, were repeated after the third and sixth cycle and after discontinuation of therapy. Response was evaluated using WHO criteria (World Health Organisation, 1979). A complete response (CR) required the disappearance of all known disease, determined by two observations not less than 4 weeks apart, and for patients with the primary tumour in place an endoscopic confirmation of a complete response with normal endoscopic biopsies. A partial response was defined as a decrease by at least 50% reduction in the sum of the products of the largest perpendicular diameters in all measurable lesions or at least a 30% reduction of the largest diameters in uni-dimensional disease (evaluable disease) for at least 4 weeks. It is not necessary for all lesions to have regressed to qualify for partial response, but no lesion should have progressed and no new lesion should appear. Stable disease was defined as less than 50% decrease and less than 25% increase in tumour size. Progressive disease was a greater than 25% increase of one or more measurable lesions or the development of new lesions.

The duration of response was defined as lasting from the start of treatment to documentation of the disease progression. Patients with stable disease received up to a maximum of six cycles of treatment. In patients achieving a partial or complete response, an additional two cycles were allowed. Patients were followed for survival and disease progression every 3 months until death.

### Statistical considerations

Patient enrolment followed a five-step sequential design. If no response was seen in the first eight patients further accrual had to be halted. Otherwise an additional six patients could be entered and if at least two patients responded again six patients had to be entered. In the fourth step 10 more patients were entered if at least four responses were observed in the 20 patients that were treated. Finally when 30 patients were treated the trial was continued with an additional 20 patients if the observed number of responses was at least 50%. Under this design there is only an 18% chance of continuing the trial while the true response percentage is below 40%.

Actuarial survival was calculated using the method of Kaplan and Meier.

## RESULTS

Fifty-one patients were entered in this study. Patient characteristics are listed in Table 1

**Table 1:**
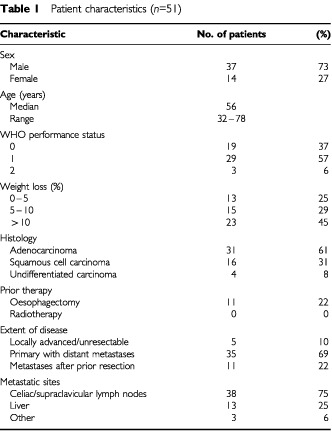
Patient characteristics (*n*=51)

. All patients received at least three cycles of chemotherapy and all were evaluable for toxicity and response. A total of 286 cycles were administered (median six, range 3–8). Nine patients received only three cycles. Three of these nine patients had progressive disease and in five patients with stable disease who had persistent dysphagia treatment was stopped and these patients were palliated by oesophageal stenting. One patient refused further treatment. Five of the remaining 42 patients did not complete six cycles of therapy. Two patients were not able to continue treatment after four cycles for reasons of toxicity mainly consisting of fatigue, one patient developed a cerebrovascular accident and two patients had progressive disease after the fourth and fifth cycle, respectively. Seven patients who had achieved a partial response received eight cycles of treatment.

Seventy-one chemotherapy cycles (25%) were delayed. In 19 (37%) patients there was no treatment delay; one, two or more delays were required in respectively seven (14%), 11 (22%) and 14 (27%) patients. Sixty-five cycles in 25 (49%) patients were delayed for 1 week because of a granulocyte count <0.75×10^9^ l^−1^. Four cycles were delayed for 1 week because of infections without neutropenia, one cycle was delayed for 1 week because of elevated liver-enzymes due to co-medication and one cycle was delayed for 3 weeks in a patient who developed a broncho-oesophageal fistula 4 days after the start of chemotherapy. The planned and achieved dose intensity were for cisplatin 30 and 26.4 mg m^−2^ per week, respectively, and for paclitaxel 90 and 79.3 mg m^−2^ per week, respectively.

### Haematological toxicity

Neutropenia grade 3 and 4 were observed in 39 and 31% of patients and in 23 and 10% of cycles, respectively. The nadir for granulocytes usually occurred after the fourth or fifth cycle of treatment. Neutropenic infections were not observed. No thrombocytopenia was seen. Red cell transfusions were administered in seven patients.

### Non-haematological toxicity

Grade 1 sensory neurotoxicity was observed in 22 patients (43%) and grade 2 in 10 patients (20%). Six of these in total 32 patients had a complete resolution of sensory neurotoxicity and in 11 patients neurotoxicity partially subsided. Five patients developed infections without neutropenia. Three of these patients were admitted because of pneumonia, an urinary tract infection and an infected subcutaneously implanted intravenous access device, respectively. All patients recovered after treatment with antibiotics. Two patients were admitted for gastro-intestinal toxicity. In total five patients (10%) were hospitalised, three patients because of treatment-related complications and two patients because of infections without neutropenia. There were no treatment-related deaths. Other toxicities were usually mild and are listed in Table 2

**Table 2:**
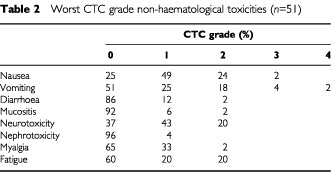
Worst CTC grade non-haematological toxicities (*n*=51)

.

### Responses

All 51 patients had measurable disease. The overall response rate was 43%; 20 patients (39%) had a PR and two patients (4%) had a CR. Stable disease was observed in 21 patients (41%) and PD in eight patients (16%). Twenty-one of 22 responding patients had bi-dimensionally measurable lesions and one patient had uni-dimensionally evaluable disease. In 15 of 22 responding patients the response was already documented after three courses of chemotherapy. The duration of complete response was 7 months in a patient with a local recurrence and lymph node metastases. The second patient who had a complete response received additional radiotherapy on the primary tumour and supraclavicular region and is still disease free 29 months after start of treatment. The median duration of response (measured from start of treatment) in the patients with a PR was 8 months (range 5–29+ months). Twenty-one patients (41%) had stable disease with a median duration of 6.5 months. After a response to chemotherapy definitive local therapy using either radiotherapy or surgery was attempted in nine patients with either locally advanced disease or lymph node metastases confined to the celiac or supraclavicular region (M1a disease). Two patients with an irresectable tumour underwent an oesophageal resection, pathologic examination of the resected specimen showed tumour free margins and these patients are disease free 20 and 28 months, respectively, after surgery. Seven patients with M1a disease received radiotherapy at a dose of 50 Gy at the primary tumour and involved lymph nodes; two of these patients are disease free after 24 and 29 months, respectively. The overall response rates for patients with adenocarcinoma, and squamous cell carcinoma were 39 and 44%, respectively, and three of the four patients with an undifferentiated carcinoma achieved an objective response.

### Survival

After a median follow-up of 32 months (range 1–32 months) 12 patients (24%) are still alive. The median actuarial survival in all patients was 9 months (range 2–29+ months), with a one-year survival rate of 43%. The median actuarial survival for responding patients is 12 months (range 6–29+ months), compared to 7 months (range 2–31+ months) in non-responding patients.

## DISCUSSION

Chemotherapy either alone or in combination with radiotherapy is frequently used preoperatively in patients with resectable disease. For patients with irresectable and/or metastatic disease chemotherapy may offer a chance of both tumour regression and palliation of symptoms. The effect of chemotherapy on survival in this group of patients is unclear due to a lack of randomised phase III studies comparing chemotherapy to best supportive care.

The combination cisplatin and 5-fluorouracil is probably the most frequently used combination in the treatment of oesophageal cancer. Pre-operative treatment with this combination was tolerated well by patients with resectable disease in two large randomised trials (Kelsen *et al*, 1998; Clark, 2000). However in one of the few randomised studies performed in patients with metastatic disease, the toxicity of this regimen appeared to be severe (Bleiberg *et al*, 1997). In that trial 88 patients with metastatic oesophageal cancer received either cisplatin in combination with 5-fluorouracil or cisplatin alone. In the cisplatin/5-fluorouracil arm there were 16% treatment related deaths, mostly due to neutropenic sepsis, *versus* 0% in the cisplatin arm. Because of this high incidence of treatment-related deaths the higher response rates observed in the cisplatin/5-fluorouracil arm most likely did not translate in a significant survival benefit compared to treatment with cisplatin alone. The difference in tolerability of chemotherapy between patients with resectable disease and patients with metastatic disease could be explained by the fact that patients with metastatic disease often have an impaired performance status, substantial weight loss and co-morbidity.

Recently, several new agents such as the taxanes, irinotecan and vinorelbine, have shown promising activity in the treatment of oesophageal cancer. A further advantage of these new agents is that they cause less mucosal toxicity compared to the combination of 5-fluorouracil and cisplatin with or without leucovorin.

In a previous study we demonstrated the feasibility of cisplatin and paclitaxel administered in a treatment interval of 2 weeks (van der Gaast *et al*, 1999). We were able to decrease the treatment interval because we retreated the patients when their granulocytes were above 750×10^6^ l^−1^ instead of the more common used threshold for retreatment of 1500×10^6^ l^−1^. The safety of this approach was confirmed in the current study. Despite the fact that 70% of patients developed grade 3 or 4 neutrocytopenia, we did not observe any episode of neutropenic fever. The achieved dose intensity was for cisplatin 26.4 and for paclitaxel 79.3 mg m^−2^ per week.

Given the fact that most patients had metastatic disease the treatment was well tolerated. Only five patients (10%) were hospitalised, three patients because of treatment-related complications and two patients because of infections without neutropenia. In general gastro-intestinal toxicity was mild and grade 2 mucositis was observed in only two patients. Sensory neurotoxicity was the predominant non-haematological side-effect: grade 1 and 2 occurred in, respectively, 43 and 20% of patients. In 19% of patients with neurotoxicity we observed a complete resolution and in 34% a partial improvement of neurotoxicity. The neurotoxicity observed with our bi-weekly regimen is comparable to the neurotoxicity observed with regimens of cisplatin and paclitaxel administered every 3 weeks. This may partly be explained by the fact that neurotoxicity due to cisplatin is more correlated with the cumulative cisplatin dose than with the dose-intensity (Hilkens *et al*, 1995). The median cumulative dose of cisplatin in our study was only 360 mg m^−2^.

The combination of cisplatin and paclitaxel with or without 5-fluorouracil was tested in patients with oesophageal cancer in three other studies. Ilson *et al* (1998) treated 61 patients with the combination of paclitaxel 175 mg m^−2^ administered over 3 h on day 1, cisplatin 20 mg m^−2^ days 1–5 and 5-fluorouracil 1000 mg m^−2^ days 1–5; 48% of the patients had to be admitted for reasons of toxicity. In a subsequent study 5-fluorouracil was omitted and paclitaxel 200–250 mg m^−2^ was administered over 24 h followed by cisplatin 75 mg m^−2^ (Ilson *et al*, 2000). Cycles were repeated every 3 weeks and all patients received granulocyte colony stimulating factor support. The toxicty in this study was also considerable and 50% of the patients had to be hospitalised due to toxicity and 11% of the patients died from treatment-related complications. In our study we used, expressed as administered dose per week, a comparable dose of paclitaxel and a higher dose of cisplatin but observed no severe toxicity. The fact that we administered paclitaxel over 3 h instead of 24 h might explain this difference (Eisenhauer *et al*, 1994). Petrasch *et al* (1998) treated 24 patients with cisplatin 50 mg m^−2^ and paclitaxel 90 mg m^−2^ (3-h infusion), also administered every 2 weeks. Using this, although compared to our study, lower dose of paclitaxel they also observed no major toxicities.

The response rate of 43% with a median duration of response of 8 months observed in our study is in line with the results reported in the other studies with cisplatin and paclitaxel however with substantial less toxicity. Of the 20 responding patients four patients, who received additional radiotherapy or surgery, are still disease free after a follow-up of 20–29 months.

In most studies on neo-adjuvant chemotherapy in patients with oesophageal cancer response to chemotherapy is an important prognostic factor. The tolerability of this bi-weekly regimen and the high response rate observed in this study renders it attractive for use in a neo-adjuvant setting. One of the reasons for the negative results of the Intergroup trial (Kelsen *et al*, 1998) comparing chemotherapy followed by surgery to surgery alone in 440 patients with resectable adenocarcinomas or squamous cell carcinomas, might be the low response rate of 19% obtained with the combination of cisplatin and 5-fluorouracil. Contrasting the negative results of the Intergroup study are the results of a recently reported Medical Research Council (MRC) trial (Clark, 2000) randomising 802 patients to two pre-operative cycles of cisplatin and 5-fluorouracil followed by surgery or surgery alone, as well as one of our own previous studies randomising 163 patients to pre-operative treatment with cisplatin and etoposide followed by surgery or surgery alone (Kok *et al*, 1997). In both studies survival was significantly better in patients receiving pre-operative chemotherapy. Chemotherapy before surgery is therefore still an option for patients with resectable oesophageal cancer. A randomised study investigating the efficacy of this bi-weekly cisplatin/paclitaxel regimen as a pre-operative treatment would therefore be appropriate.

In conclusion, cisplatin and paclitaxel administered every 2 weeks is an active combination in the treatment of patients with advanced oesophageal cancer. The toxicity profile of this regimen compares favourable to that of previously used cisplatin-, and paclitaxel-based regimens. Trials are underway evaluating this bi-weekly regimen in a neo-adjuvant setting.
